# The *Ashtavaidya* physicians of Kerala: A tradition in transition

**DOI:** 10.4103/0975-9476.74424

**Published:** 2010

**Authors:** Indudharan Menon, Annamma Spudich

**Affiliations:** *National Center for Biological Sciences, Tata Institute of Fundamental Research, Bangalore, India.*

**Keywords:** *Ashtavaidya*, Gurukulam learning, Indian sciences, Kerala Ayurveda, Traditional medicine

## Abstract

This paper presents what we have learned from the *Ashtavaidya Ayurveda* physicians of Kerala regarding the status of their unique medical tradition of Ayurveda in the contemporary context. We extensively interviewed several practicing *Ashtavaidyas* for the *“Living History of Indian Scientific Traditions”* archive, a new initiative at the NCBS, Bangalore to study the history of Indian sciences. As heirs of a tradition that has adapted and evolved over centuries without compromising its fundamental principles, their views on Ayurveda presented here represent an important contribution to the current debate on the role of traditional medicine in the Indian public health system.

## INTRODUCTION

Traditional medical systems remain important resources of healthcare worldwide in spite of increasing access to modern biomedicine. Selected techniques of traditional medical practices are part of the health regimen of people throughout the world. With increased interest and access to healing practices that are radically different from modern biomedicine, critical questions regarding the validity and efficacy of such practices are being raised.[1,2] We have observed that abridged and simplified versions of Ayurvedic therapeutic methods are supplanting age-old, established procedures, and that such simplified versions are gaining popularity throughout the world. Traditional diagnostic methods are being abandoned and modern biomedical terms and techniques of analysis are replacing them in the day-to-day practice of Ayurveda. Major changes are also taking place in the educational system and transmission of Ayurvedic knowledge in order to comply with the norms of modern biomedicine. Given that such transformations are happening to this traditional medical culture, the time is appropriate to examine and evaluate the consequences of such modernization to the integrity and future of traditional medical systems, and their validity in general and Ayurveda in particular, and their place in contemporary health care.

We present here what we learned regarding these transitions from *Ashtavaidya* Ayurveda physicians of Kerala whom we interviewed for the *“Living History of Indian Scientific Traditions”* archival project at the NCBS, Bangalore. *Ashtavaidya*scholar physicians have contributed significantly to Kerala’s reputation as the preeminent center for Ayurveda in India. Their unique methods of healing, while based on the classical textual tradition of Ayurveda, have been enriched over centuries through interactions with folk medical practices of Kerala. Today, their traditional culture and practice of Ayurveda are at a crossroads as they are obliged to comply with norms imposed by modern medicine, and adapt to the changed socio-economic context. As heirs of a tradition that has evolved over centuries without compromising its fundamental principles, the perspectives of the *Ashtavaidyas* are vital for the future of traditional medicine.

## THE INTERVIEWS

During 1997-2001, we held several conversations with two of the *Ashtavaidyas* of Kerala, Olassa Chirataman Narayanan Moos and Vayaskara Aryan Moos. These interviews highlighted the urgency of documenting this scholar physician tradition at a crucial stage in its history. In 2009, with support from the National Centre for Biological Sciences/TIFR, Bangalore, extensive interviews were conducted with Vaidyamadham Cheriya Narayanan Namboodiri, the doyen of the remaining *Ashtavaidyas* of Kerala, as well as practicing physicians of his family. Vaidyamadham Namboodiri is a unique repository of Ayurveda theory and practice, and the history and lore of the *Ashtavaidya* tradition. We also interviewed Alathiyoor Narayanan Nambi and Plamanthol Shankaran Moos, practicing physicians of two legendary *Ashtavaidya families* whose ancestors through their writings have played a major role in disseminating the classical knowledge of Ayurveda.

The interviews record, so to speak, from the mouth of the gurus (*gurumukham*), their education and training, the special practices of each lineage, issues facing traditional medical practice, and their perspectives on the culture of modern Ayurveda. This paper provides a bird’s-eye view of this ancient and scholarly Indian medical tradition from some of its most recent practitioners, and their concerns about the future of their ancestral therapeutic techniques and method of learning. We also present here their views regarding a revised system of Ayurvedic medical training incorporating aspects of the classical scholarship that constitutes the hallmark of the *Ashtavaidyas*.

## AYURVEDA TRADITION OF KERALA

Folk healers of the sub-continent, healing practices in the Vedas, and Buddhism, Jainism and other ascetic and philosophical traditions have all contributed to the evolution of classical Ayurveda.[3] The three canonical texts of classical Ayurveda, *Carakasamhita, Susruthasamhita* and *Ashtangahrdayam,* reveal such a multi-cultural origin. For example, *Sushrutasamhita* exhorts that *“knowledge of medicinal plants and their identification should be gained with the help of cowherds, hermits, hunters, forest-dwellers and those who gather plants of the forest for food”*.[4] Over the centuries Ayurveda has remained open to new healing methods brought by immigrants, particularly from the Persian and Arab schools.

Kerala, with its abundant resource of medicinal plants, has a long history of folk medical traditions practiced by healers from all levels of society. The arrival in Kerala of the canonical *Ashtangahrdayam* composed between the 6^th^and 7^th^century CE by Vagbhata, a Buddhist from Sind, stimulated the development of a new dynamic medical culture. Certain upper-caste Sanskrit-literate healers of Kerala adopted this work as their source book while continuing to draw on regional folk and physical medical practices from diverse sources such as poison therapy and Kalaripayath, the martial arts of Kerala.

## ON THE ASHTAVAIDYA AYURVEDIC TRADITION

Between the 13^th^and the 17^th^centuries, with generous royal and individual patronage, a fertile intellectual milieu developed around temples in Kerala, especially in the Nila valley region in Malabar, where scholarship and scientific research on medicine, mathematics and astronomy made significant progress. The *Ashtavaidya* culture evolved in this environment, blending the Ayurveda of *Ashtangahrdayam* with the knowledge and practices of local healers.

Among the healers of Kerala, the *Ashtavaidyas* represent the Brahmin scholar physicians who were masters of the eight branches (Ashtanga) of Ayurveda mentioned in classical texts. *Ashtangahrdayam,* the primary text of the Ashtavaidyas [Table 1], deals with these eight branches of therapy.

**Table 1:** The eight branches (ashtanga) of classical Ayurveda[5]

Kaya	General medicine mainly dealing with digestive disorders
Bala	Pediatrics including obstetrics
Graha	Psychological disorders due to possession by evil spirits etc.
Urdhvanga	Diseases of the head (eyes, ears, nose, throat and teeth)
Shalya	Surgery and treatment for external injuries
Damshtra	Toxicology (treatment for poisoning, snake and insect bites)
Jara	Geriatrics and rejuvenation
Vrisha	Aphrodisiacs and treatment for sterility

According to tradition, initially eighteen upper caste families of Kerala were designated as *Ashtavaidyas*. Each *Ashtavaidya* family developed its own therapeutic specialties and its specific methods of transmission. Although many of the specialties were guarded as family secrets, students outside the family were accepted as disciples. This helped disseminate their knowledge beyond the family circle and create new lineages of transmission. The *Ashtavaidyas* have enriched Ayurvedic literature through their Sanskrit commentaries on the *Ashtangahrdayam*such as *Hrdayabodhika* and *Vakyapradipika,* and compendiums in Malayalam such as *Alattur Manipravalam,*[6] *Cikitsamanjari, Sahasrayogam and Sindhuramanjari*.[7] *Ashtavaidyan*Vayaskara N.S.Moos made one of the most significant contributions to 20^th^century Ayurvedic literature by publishing ancient texts[8] and his own original works. More recently, Vaidyamadham Namboodiri has written books and over a hundred newspaper articles to inform the public about Ayurveda. Today, only a handful of *Ashtavaidya*physicians trained in their ancestral system of study by apprenticeship remain in practice and the tradition itself is at a crucial turning point.

## CLASSICAL GURUKULAM EDUCATION OF AN AYURVEDIC PHYSICIAN

The senior *Ashtavaidya*physicians we interviewed were trained in the *Gurukulam* system and had practiced for more than 40 years. They were masters of healing with deep knowledge of classical texts and all aspects of traditional therapy. All belonged to families where, in the words of Vaidyamadham Namboodiri, *“we lived and breathed Ayurveda from birth.”* In general the methods and the progression of their training are those followed for centuries for mastering any Shastra or body of knowledge in India.[9,10]

The education of a traditional *Ashtavaidya*in the Gurukulam system involved a long period of intense study and apprenticeship under accomplished masters. Knowledge of Sanskrit in all its complexity through the study of grammar, poetry and drama was considered essential to decipher and analyse the intricate and implied meanings in the ancient medical texts. In addition, the students mastered Sanskrit works on *Tarka* (the rules of reasoning and argument), and the traditional philosophies of *Nyaya, Vaisheshika* and *Samkhya*. In the words of *Ashtavaidyan* Olassa Narayanan Moos, such erudition was necessary to become *agaadha panditas,* profound scholars, of Ayurveda.

The *Ashtavaidyas* emphasised that a solid foundation in the *Ashtangahrdayam* of Vagbhata is a prerequisite for practicing their style of Ayurveda. Therefore, *Ashtavaidyas* began the study of Ayurveda by memorizing all 7120 odd verses of the *Ashtangahrdayam*. According to Vaidyamadham Namboodiri this text “ought to be worn around the neck as a garland” and readily available “in the throat” (*kanthastham*) for immediate recall.

Traditionally, during the period of apprenticeship under a guru, student physicians wrote out the prescription for patients. The master would only recite the first few words of the verse that contains the medicinal formulation he wished to prescribe and the students were expected to add the rest of the verse from memory and complete the prescription.

With the textual source at the tip of his fingers (or in his throat!), the apprentice, by observing his master practicing his art, gradually understood the rationale behind the choice of each treatment, for*“it is through persevering practicing (under the guidance of a master) that one attains the clear vision capable of rendering one’s treatment efficacious.”*[11]

Although ideally the number of years of education was said to be *“five years of textual study, five years of learning about medicinal plants in the forest, and five years of apprenticeship at home”*(in Malayalam *ezhittil anju, kattil anju, veettil anju*), in practice the number of years of learning medical texts started in the mid-teens and continued until the mid-twenties. Through his years spent in the study of Sanskrit language and literature, followed by classical texts of Ayurveda, a qualified *Ashtavaidya* learned to provide truly individualized therapy, the hallmark of their tradition. The students were also taught to identify plants for making personalised medicinal preparations by varying the ingredients appropriate to each patient’s ailment.

Individualized treatments taking into account all aspects of a patient’s life are a specialty of traditional Ayurveda. The following verses from the *Ashtangahrdayam*, emphasize the importance of examining in minute detail the personal history of a patient before diagnosing the disease and prescribing a treatment. *“The physician who minutely and attentively examines the condition of the vitiated tissues and waste products, the environment in which the patient lives, the patient’s general vitality, the season, the patient’s digestive power, his natural constitution, his age, his state of mind, his habituations, his food habits and the stages of the manifestation of the disease, and then determines the nature of the aggravated dosha and the appropriate medication will never go wrong in the choice of treatment*.”[12]

In spite of the trend in modern India to prescribe standardized Ayurvedic medicines manufactured by pharmaceutical companies, the *Ashtavaidyas*we interviewed continue to make the effort to practice their ancestral tradition of person-centered therapy using medicines they themselves prepare that are adapted to the needs of each individual patient.

*Ashtavaidya* gurus inculcated in their disciples the view that investigating and understanding the fundamental nature of human beings, the environment in which we live, and the nature of diseases, are essential to become a master physician. Training to be a physician meant training the mind to be both analytical and intuitive, while remaining grounded strongly in factual knowledge. The Ashtangahrdayam urges the physician to adapt the rationale of existing therapies to newly emerging health issues, saying that “he *should not be ashamed because he is not able to give a name to an illness, for there is no rule that every disorder has to have a name.”*[13] In the words of one of the interviewees (Olassa Narayanan Moos), becoming a competent physician was the responsibility of both the teacher and the student, and only he who has received permission *“to practice medicine from his guru deserves the title of Bhishak or physician.”*[14] The senior physician took his role to be the guru of his lineage of disciples as a serious mandate. The preparation and use of the class of powerful single herb remedies known as *Ottamoolis* in Malayalam, were transmitted to deserving students only at the discretion of the guru. In the hands of an accomplished Vaidya, when used appropriately, even ordinary substances found in the patient’s environment can become potent medicines. And stories of miraculous cures using novel methods and unusual substances as medicine are legion among the *Ashtavaidyas*. It is said in the *Ashtangahrdayam that“there is nothing in this universe that is not a medicine and that can not be used for many purposes and in many different ways.”*[15]

Ayurveda is not only a system of healing but also a way of life. In order to develop the clarity of mind necessary to be a man of knowledge, methods of spiritual training known as *sadhana* have been an integral part of the process of learning traditional sciences in India. So, along with the formal study of texts, a period of intense spiritual training was an integral part of the formative years of *Ashtavaidya* physicians. A young physician was expected to consecrate a period of a year or more, called *bhajana,* exclusively to such practices and to the recitation of the *Ashtangahrdayam* in the family temple.

At the conclusion of his studies the novice physician transcribed a complete copy of the *Ashtangahrdayam* as an offering to his guru, cementing his relationship with the text and his teacher.

## PERSPECTIVES OF THE ASHTAVAIDYAS ON CONTEMPORARY AYURVEDA

Given the culture of intense learning they underwent to become physicians, the *Ashtavaidyas* we interviewed expressed concerns about many of the new trends in Ayurveda practice and teaching. While they are aware that Ayurveda has evolved over millennia, and that the intense training they underwent cannot be sustained in the contemporary environment, they felt that many of the modifications occurring today undermine the very nature of Ayurveda.

Traditional Ayurveda uses a vocabulary derived from the language of our everyday experience. It explains and diagnoses diseases using terms from our sensory experience such as hard, soft, moving, still, hot, cold, dry, humid, salty, bitter etc. Food and medicinal substances and their application and efficacy are also understood using similar frames of reference. Nevertheless, today the overwhelming trend is to integrate Ayurveda within the concepts and language of modern biomedicine. Since language influences our vision and understanding of the world as well as our praxis, and Ayurveda has its own vocabulary, traditional Vaidyas feel that by depending on modern biomedical terms and tools to define disease and treatment there is a danger of progressively losing the fundamental spirit of Ayurveda’s methodology. Therefore, the challenge today is to find pertinent ways to adapt Ayurveda to the modern world without it being overshadowed by conceptual frameworks alien to it.

## ON MODERN AYURVEDA EDUCATION

The *Ashtavaidyas* we interviewed were well acquainted with the present-day Ayurveda educational system since younger members of their families who had chosen to become Ayurvedic physicians were all trained in modern Ayurvedic colleges. They are deeply committed to maintaining the scholarship and the therapeutic techniques of their ancestral heritage in the modern education system, and their general observation was that attempts to develop a modern system of education for Ayurveda, with curriculums integrating methods of biomedical training, poses many challenges. They were not opposed to the idea of students learning aspects of biomedical sciences once they were well grounded in the Ayurvedic tradition. Although the current educational system produces large numbers of practitioners with a basic level of competence, the training was not deemed individual or intensive enough to provide the mastery of Ayurveda that characterized the scholar physicians of the past. In classical Ayurvedic training teachers and disciples interacted intensely on a one-to-one basis. During the long years of apprenticeship under a guru, they thoroughly studied the classical texts, aspects of medical practice such as diagnosis, preparation and administration of medicines and the philosophical and spiritual foundations of the tradition.

Another issue the *Ashtavaidyas* raised was the inadequate preparation and motivation for intense learning of students entering the Ayurveda education program. Focused preparatory studies, somewhat akin to what pre-biomedical students undergo, should be made a prerequisite for admission and would help select students with the deep motivation necessary to undertake years of scholarly study. From the start, students should have the level of competency in Sanskrit necessary for understanding linguistic nuances of classical Ayurveda texts. One of our interviewees who teaches in an Ayurvedic educational institution observed that although the curriculum covers a vast range of topics, at the completion of their studies most students had a hazy view of Ayurveda and were not prepared enough to take full advantage of the depth of Ayurvedic therapeutics. One reason for this may be that most students had no familiarity with the basic principles of traditional Indian scientific thought and found it difficult to assimilate the classical Ayurvedic mode of thinking.

According to the *Ashtavaidyas* the current education system is too narrowly focused. Of the extensive pharmacopeia of Ayurveda only a fraction is being taught, largely because importance is now given to a relatively limited number of commercially available prepared formulations. This is also largely true of the therapeutic techniques of Ayurveda. Popular and lucrative techniques are being taught and many of the classical practices are now known only to a small number of skilled older practitioners.

The practice of Ayurveda in the modern context is developing at two levels. On the one hand, a small number of Ayurvedic practitioners and Ayurveda centers treat illnesses using classical therapeutic techniques and medicinal preparations, complying with modern standards required by government. On the other hand, increasing numbers of Ayurveda-based practitioners and centers propose so-called Ayurvedic rejuvenation therapies along with a smattering of yoga, meditation and Indian philosophy. While there is room for both types of practices in the contemporary context, the distinction between the two is becoming blurred, and the economically lucrative leisure aspect is coming to represent Ayurveda as a whole. Understandably more students are embarking on the study of Ayurveda with *leisure medicine* as their goal, and the in-depth study and practice of classical Ayurveda is not finding as many devotees. This trend could have serious impact on the future of the transmission of classical Ayurvedic knowledge and therapeutic techniques.

The *Ashtavaidya* physicians we interviewed made a strong case for reestablishing the opportunity for at least a small number of students to study classical Ayurveda in depth in order to perpetuate the tradition of scholar physicians. For centuries patronage played important roles in maintaining the *Ashtavaidya* tradition. Traditionally the eldest sons of *Ashtavaidya* families had the responsibility of keeping the family tradition alive by profoundly studying Ayurveda and dedicating their lives to the practice of their ancestral method of healing. It was customary for the *Ashtavaidyas* not to accept payment for treatments and most often patients offered token gifts in kind to show their appreciation. In recognition of their service to the community and their status as scholars of Ayurveda, royal patronage, and in some cases land grants, provided them leisure to pursue their scholarly life. Today in the absence of such support the years of commitment and study required have become a luxury while many financially lucrative alternatives tempt even the most dedicated student. So adequate guaranteed financial support for students with the aptitude and motivation to undergo intense training to become scholar physicians, as is now available for advanced study in the basic sciences, would go a long way to keep this scholarly Ayurveda tradition alive.

## ON THE NEED FOR BETTER PATIENT EDUCATION

The *Ashtavaidyas* spoke of the need for educating the public about the basics of Ayurveda therapeutics. According to the *Ashtangahrdayam,*[16] successful treatments have four constituent limbs (*pada*), the physician, the medicines, the attendant (nurse) and the patient. The patient’s compliance with Ayurveda regimens requires a basic understanding of the contexts in which traditional medicine and modern biomedicine can be effective. A patient who is well informed can participate actively and accelerates the healing process.

In general, traditional Ayurvedic therapies, because of the types of procedures and formulations used, require more time to manifest their beneficial effects. Purified single molecule drugs used in biomedicine are effective within a shorter period of time, and for certain illnesses biomedicine is the only effective therapy. However, many biomedical drugs may develop adverse side effects after prolonged use, and for many ailments that require long-term therapy, traditional medicines may have advantages. Therefore, patients need to be educated about the risks and advantages of each system, like speed and efficacy versus issues of long-term side effects. The *Ashtavaidyas* consider that for certain ailments, such as those related to the locomotor, immunity and digestive systems, their methods can be more efficacious. Physicians who understand the potential of both modern biomedicine and traditional therapies should assist patients to choose the most appropriate therapy. At present the majority of patients choose traditional medicine when biomedical therapies have failed and the ailments are almost incurable, and this negatively influences the way the efficacy of traditional therapies is perceived by the public.

Throughout the world traditional medical systems are becoming part of mainstream healthcare. India is one of the few countries where different medical traditions have coexisted for centuries. The *Ashtavaidyas* argued for a novel Integrative Medicine paradigm where traditional Indian medical systems and modern biomedicine work together on an equal footing in a cooperative medical culture to form a dynamic healthcare system. With centuries-old indigenous medical systems and a strong presence of modern biomedicine, India is uniquely placed to pioneer such a medical revolution.
